# Comparative study of liposomal amphotericin B, posaconazole, and micafungin for primary antifungal prophylaxis in pediatric patients with acute leukemia

**DOI:** 10.1007/s00432-025-06289-5

**Published:** 2025-08-23

**Authors:** Anna Sophia Gottschlich, Jana Ernst, Till Milde, Bernd Gruhn

**Affiliations:** 1 Department of Pediatrics, Jena University Hospital, Jena, Germany; 2 Comprehensive Cancer Center Central Germany (CCCG), Jena, Germany; 3 Hopp Children’s Cancer Center Heidelberg (KiTZ), Heidelberg, Germany; 4 Clinical Cooperation Unit Pediatric Oncology, German Cancer Research Center Heidelberg (DKFZ), Heidelberg, Germany

**Keywords:** Primary antifungal prophylaxis, Invasive fungal disease, Acute leukemia, Liposomal amphotericin B, Posaconazole, Micafungin, Pediatric

## Abstract

**Purpose:**

Invasive fungal diseases (IFDs) are a significant cause of morbidity and mortality in pediatric patients with hematologic malignancies including acute leukemia. Our study aimed to compare the efficacy of liposomal amphotericin B (L-AMB), posaconazole or micafungin as primary antifungal prophylaxis (PAP) in pediatric patients with acute leukemia.

**Methods:**

This retrospective observational study enrolled 95 pediatric patients with acute lymphoblastic leukemia (n = 70) or acute myeloid leukemia (n = 25), undergoing chemotherapy, including those undergoing allogeneic hematopoietic stem cell transplantation at the Department of Pediatrics, Jena University Hospital, Jena, Germany. PAP regimens included L-AMB (1 mg/kg/day or 3 mg/kg twice weekly, intravenously), posaconazole (100–300 mg/day, according to blood concentration, orally or intravenously) and micafungin (1 mg/kg/day or 3 mg/kg twice weekly, intravenously). Thirty-four patients (35.8%) received L-AMB, 37 patients (38.9%) received posaconazole, and 24 patients (25.3%) received micafungin. Patients with a history of IFD or concurrent or changing PAP were excluded. The primary endpoint was the occurrence of breakthrough IFD, while secondary endpoint included IFD-free survival. Statistical analyses were performed using Kaplan–Meier survival analysis, Gray’s test and Cox regression to evaluate IFD-free survival.

**Results:**

The overall incidence of IFD was 14.7% (14 of 95 patients). IFD developed in 10 of 33 patients (29.4%) receiving L-AMB, in 4 of 38 (10.8%) patients receiving posaconazole and in none of the patients receiving micafungin. IFD-free survival was 70.6% in the L-AMB group, 89.2% in the posaconazole group and 100% in the micafungin group (*p* = 0.005, log-rank test). Significant differences were also observed in the cumulative incidences of breakthrough IFDs (*p* = 0.006) assessed by Gray’s test. In multivariate Cox analysis, dichotomized prophylaxis regimes (posaconazole or micafungin vs. L-AMB) were independently associated with a reduced risk of IFD (HR = 0.244; 95% CI 0.076–0.777; *p* = 0.017). Age ≥ 10 years predicted inferior IFD-free survival (HR = 3.665; 95% CI 1.224–10.980; *p* = 0.020).

**Conclusion:**

We found a significant difference in efficacy between the three antifungal prophylaxis regimens. In our study, micafungin achieved the lowest IFD breakthrough rate. However, multicenter clinical studies would be needed to confirm the results.

## Introduction

Invasive fungal diseases (IFDs) are a significant cause of morbidity and mortality in pediatric patients with hematologic malignancies, including acute leukemia (Groll et al. 2014). Children diagnosed with acute myeloid leukemia (AML) or those who experience long-lasting neutropenia (< 500/μL for > 7 days) are at particularly high risk of developing IFDs (Stemler et al. 2023). Acute leukemia, encompassing both acute lymphoblastic leukemia (ALL) and AML, is the most common hematologic malignancy in children (Spix et al. 2023). Therefore, these diseases represent the primary focus of this study.

The most common pathogens responsible for IFDs in this patient group include yeasts from the *Candida* species and molds, particularly *Aspergillus* spp. (Groll et al. 2014; Maschmeyer et al. 2007). Recent data from a multicenter study involving 62 pediatric oncology centers in Germany, Austria, and Switzerland reported an IFD incidence of 4.6% among all pediatric oncology patients in 2023 (Seidel et al. 2025), highlighting the continued relevance of effective antifungal prophylaxis in this population. In contrast, other studies focusing specifically on children with acute leukemia have reported higher incidence of IFDs, ranging between 5 and 18% with a peak of up to 24% in AML patients (Gal Etzioni et al. 2024; Giacchino et al. 2011). Mortality rates associated with these infections are even higher, ranging from 17.5 to 68% in patients with acute leukemia (Gal Etzioni et al. 2024) and from 35 to 41% in children undergoing hematopoietic stem cell transplantation (HSCT) (Robenshtok et al. 2007).

The use of primary antifungal prophylaxis (PAP) has significantly reduced the incidence of IFDs in children with acute leukemia (Lehrnbecher et al. 2020). A meta-analysis from 2018 showed that liposomal amphotericin B (L-AMB), posaconazole and micafungin had lower rates of breakthrough IFD compared to a placebo (Lee et al. 2018), all of which were compared in this study. Regarding the specific recommendations for the three antifungal agents, there is limited pediatric-specific data, though general guidelines for adults are often applied to children as well (Maertens et al. 2022). Historically, L-AMB was the preferred agent for patients with ALL. However, its use has declined due to its toxicity profile and the availability of safer and more effective alternatives (Stemler et al. 2023). L-AMB is available only as an intravenous formulation. Posaconazole is widely recommended for prophylaxis in patients with AML, high-risk neutropenia, or post-HSCT, with the strongest evidence supporting its use in preventing invasive *Aspergillus* infections, particularly in patients with graft-versus-host disease (GVHD) (Stemler et al. 2023; Ullmann et al. 2016). Studies suggest that a minimum trough level of 500 ng/mL is required to achieve effective prophylaxis (Andes et al. 2009), with some studies recommending even higher levels, such as 700 ng/ml (Jang et al. 2010). It is available in multiple formulations including oral suspension, delayed-release tablets and intravenous (IV) solutions, providing flexibility in administration depending on patient needs and clinical settings (Groll et al. 2020). Micafungin, an echinocandin, represents a newer option in this context. It is exclusively available as an intravenous formulation and has shown promising results in pediatric patients, though data remain limited to small-scale studies (Stemler et al. 2023).

## Patients and methods

### Study design and patients

This retrospective observational study was conducted at the Department of Pediatrics, Jena University Hospital, Jena, Germany, and aimed to evaluate antifungal prophylaxis regimens in pediatric patients with a confirmed diagnosis of ALL or AML. The study included patients who underwent chemotherapy, including those undergoing allogeneic HSCT, and who received PAP between January 2008 and December 2023. Specifically, patients were treated with one of the following antifungal agents: L-AMB, posaconazole or micafungin. L-AMB was given intravenously at a dosage of 1 mg/kg/day (inpatient setting) or 3 mg/kg twice weekly (outpatient setting). Posaconazole was administered orally or intravenously at a dosage of 100–300 mg/day aiming for a trough level above 700 ng/mL. Micafungin was given intravenously at either 1 mg/kg/day (inpatient setting) or alternatively at 3 mg/kg twice weekly (outpatient setting).

Patients diagnosed with ALL were treated according to the AIEOP-BFM ALL 2009 or AIEOP-BFM ALL 2017 protocols, or the ALL-REZ BFM 2002 protocol in case of relapse, depending on the year of diagnosis. Patients diagnosed with AML received treatment according to the AML-BFM 2004 or AML-BFM 2012 protocols. Patients with relapsed AML were treated according to the Pediatric Relapsed AML and AIEOP-BFM AML 2020 protocols. In patients with ALL, antifungal prophylaxis was administered during the induction and reinduction phase of chemotherapy. In patients with AML, prophylaxis was provided continuously throughout the entire chemotherapy treatment course. In patients undergoing HSCT, antifungal prophylaxis was given until day + 100 post-transplantation. The assignment of antifungal agents was primarily based on patients´ diagnosis. Patients with ALL predominantly received L-AMB or micafungin, while those with AML were mainly treated with posaconazole. The decision-making process remained consistent throughout the 16-year study period. Therefore, within the constraints of diagnosis-based treatment allocation, the choice of antifungal agent can be considered as quasi-random.

Notably, patients undergoing allogeneic HSCT were treated in rooms with laminar airflow and high-efficiency particulate air (HEPA) filtration. All other patients were treated in standard patient rooms, with thorough education on hygiene and fungal exposure precautions. Induction chemotherapy for patients with ALL was administered during inpatient hospitalization, subsequent treatment phases were performed on an outpatient basis. None of the included patients had relevant comorbidities such as pre-existing or therapy-induced diabetes mellitus that could have influenced susceptibility to IFD. Patients with a documented history of IFD or those receiving concurrent treatment with multiple antifungal agents were excluded from the analysis.

#### Definitions

PAP refers to the preventive use of antifungal drugs to reduce the risk of IFDs in high-risk patients, such as those undergoing chemotherapy for leukemia. According to established definitions (e.g., Groll et al.), patients considered at high risk for IFD include those with AML, relapsed acute leukemia, recipients of hematopoietic stem cell transplantation (HSCT), and patients with high-risk ALL (Groll et al. 2021). Although patients with standard-risk ALL are generally considered at lower risk, antifungal prophylaxis was routinely administered to all patients with ALL at our center due to local conditions, specifically prolonged and repeated construction work in the hospital during the study period, which posed an increased risk of environmental fungal exposure. IFDs were classified according to the European Organization for Research and Treatment of Cancer (EORTC) criteria (Donnelly et al. 2020). We included “possible” IFD cases in line with the updated EORTC definitions and to remain consistent with other recent real-world studies assessing antifungal prophylaxis. Especially in our setting, with heterogeneous risk groups and periods of increased environmental exposure, these cases were considered clinically meaningful. Excluding them would likely lead to an underestimation of the true IFD burden.

#### Endpoints

The primary endpoint was the occurrence of breakthrough IFDs. Secondary endpoint included IFD free-survival because it provides important insights into the effectiveness of the PAP regimens.

#### Data collection

Patient data were retrospectively collected from electronic medical records. The following variables were extracted: demographic information (sex and date of birth), diagnosis details (type of leukemia and risk group), treatment information (age at diagnosis, chemotherapy protocols, date and type of transplant, antifungal agents used), plasma concentrations of posaconazole (when available), and the incidence of IFDs and death.

#### Statistical analysis

The data were initially coded in an Excel sheet before being imported into SPSS Statistics (Version 29.0.2.0) for further analysis. Descriptive statistics were used to summarize categorical variables, expressed as frequencies and percentages, and continuous variables, expressed as medians with ranges. Survival analysis for IFD-free survival was performed using the Kaplan–Meier method, with comparisons between survival curves by using the log-rank test. The cumulative incidence of IFDs was assessed using the Gray test for competing risks, conducted in R (version 4.4.2). Additionally, a multivariate analysis was performed using Cox proportional hazards regression to assess the impact of various factors, including PAP, gender, age at diagnosis, diagnosis and HSCT, on IFD-free survival. A two-sided *p*-value of < 0.05 was considered statistically significant.

## Results

### Patient characteristics

A total of 95 pediatric patients with acute leukemia were included in this study. Of these, 70 patients (73.7%) were diagnosed with ALL and 25 patients (26.3%) with AML. The cohort included 50 male (52.6%) and 45 female (47.4%) patients, with a median age at diagnosis of 6 years (range: 0.5–19 years). HSCT was performed in 48 patients (50.5%). This included 29 of 70 patients with ALL (41.4%) and 19 of 25 patients with AML (76.0%), indicating that HSCT was substantially more common among patients with AML (Table 1). Among the 95 patients, the distribution of high-risk (HR) and low-/intermediate-risk (LR/IR) subtypes was as follows: In ALL, 34 (35.85) were HR-ALL and 36 (37.9%) LR-/IR-ALL; in AML, 22 (23.2%) were HR-AML and 3 (3.2%) LR-/IR-AML. Relapse occurred in 24 patients (25.3%).

**Table 1:** Characteristics of patients (n = 95) in total and by prophylaxis group

Characteristics	Total (n = 95) No. (%)	L-AMB, (n = 34) No. (%)	Posaconazole (n = 37) No. (%)	Micafungin (n = 24) No. (%)
Median age at diagnosis (years)	6	4.5	6	6
Sex				
Male	50 (52.6)	19 (55.9)	18 (48.6)	13 (54.2)
Female	45 (47.4)	15 (44.1)	19 (51.4)	11 (45.8)
Diagnosis				
ALL	70 (73.7)	25 (73,5)	22 (59.5)	23 (95.8)
HR-ALL	34 (35.8)	14 (41.2)	14 (37.8)	6 (25.0)
LR-/IR-ALL	36 (37.9)	11 (32.4)	8 (21.6)	17 (70.8)
AML	25 (26.3)	9 (26.5)	15 (40.5)	1 (4.2)
HR-AML	22 (23.2)	9 (26.5)	12 (32.4)	1 (4.2)
LR-/IR-AML	3 (3.2)	0 (0.0)	3 (8.1)	0 (0.0)
Relapse	24 (25.3)	12 (35.3)	11 (29.7)	1 (4.2)
ALL	15 (15.8)	9 (26.5)	5 (13.5)	1 (4.2)
AML	9 (9.5)	3 (8.8)	6 (16.2)	0 (0.0)
HSCT	48 (50.5)	21 (62.8)	24 (64.9)	3 (12.5)
ALL	29 (30.5)	14 (41.2)	12 (32.4)	3 (12.5)
AML	19 (20)	7 (20.6)	12 (32.4)	0 (0.0)
IFD	14 (14.7)	10 (29.4)	4 (10.8)	0 (0.0)
Death	9 (9.5)	7 (20.6)	2 (5.4)	0 (0.0)

*ALL* acute lymphoblastic leukemia, *AML* acute myeloid leukemia, *HSCT* hematopoietic stem cell transplantation, *IFD* invasive fungal disease, *L-AMB* liposomal amphotericin B, *No.* Number

### Outcomes

Tables 1 and 2 show that 34 patients received L-AMB, 37 patients received posaconazole, and 24 received micafungin as PAP. The overall incidence of IFD was 14.7% (14 of 95 patients) (Table 2).

**Table 2:** Incidence and EORCT classification of invasive fungal diseases by prophylaxis group

Incidence of IFD	Overall (n = 95)	L-AMB (n = 34)	Posaconazol (n = 37)	Micafungin (n = 24)
overall	14	10	4	0
Possible	4	4	0	0
Probable	7	5	2	0
Proven	3	1	2	0

*IFD* invasive fungal disease, *L-AMB* liposomal amphotericin B

In the L-AMB group, 10 of 33 patients (29.4%) developed an IFD. They were classified as one proven (*Aspergillus flavus*), five probable IFDs (including *Aspergillus* and *Candida*), and four possible IFDs (Table 3). Seven infections occurred during chemotherapy, one in relapse therapy and two after HSCT. The most commonly affected organ was the lung (6/10), followed by the liver (3/10), and the colon (1/10). One patient in this group died due to IFD (Table 3).

**Table 3:** Characteristics of patients with invasive fungal disease under primary antifungal prophylaxis with liposomal amphotericin B or posaconazole

Patient	Age at diagnosis (years)	Diagnosis (ALL/AML)	IFD classification (per EORCT)	Pathogen	HSCT	Timing of IFD onset	Infection site	Time to event (days)	Death
L-AMB									
1	14	ALL	1	X	Yes	1	2	135	No
2	10	ALL	2	*Aspergillus*	No	1	1	13	No
3	2	ALL	1	X	No	1	1	32	No
4	3	ALL	2	*Candida*	No	2	3	1308	No
5	15	ALL	2	*Aspergillus*	No	1	1	423	No
6	0.25	ALL	1	X	Yes	1	1	74	No
7	13	ALL	2	*Aspergillus*	Yes	3	2	1937	No
8	17	AML	2	*Aspergillus*	No	1	1	1048	Yes
9	10	AML	1	X	Yes	1	2	141	No
10	12	AML	3	*Aspergillus flavus*	Yes	3	1	1160	No
Posacona-zole									
11	3	ALL	2	*Aspergillus*	Yes	2	1	522	No
12	17	ALL	2	*Aspergillus*	Yes	3	1	220	No
13	0.25	ALL	3	*C. albicans*	Yes	1	2	90	No
14	9	AML	3	*Aspergillus*	Yes	3	1	231	Yes

Shown are diagnosis (ALL/AML), IFD classification based on EORCT criteria (1 = possible, 2 = probable, 3 = proven), identified pathogens, timing of IFD onset during treatment (1 = during chemotherapy (before HSCT), 2 = during relapse treatment (before HSCT), 3 = post-HSCT, infection site (1 = lung, 2 = liver, 3 = colon), and outcome. “X” indicates no specific pathogen identified

*ALL* acute lymphoblastic leukemia, *AML* acute myeloid leukemia, *EORCT* European Organization for Research and Treatment of Cancer, *HSCT* hematopoietic stem cell transplantation, *IFD* invasive fungal disease, *L-AMB* liposomal amphotericin B

In the posaconazole group, 4 of 38 (10.8%) patients with IFDs were documented, including two proven cases (*Aspergillus spp., Candida albicans*) and two probable (*Aspergillus spp.*) infections (Table 3). One infection occurred during chemotherapy, one during relapse therapy and two after HSCT. Three infections involved the lung, and one involved the liver (Table 3). Notably, posaconazole was the only antifungal agent with therapeutic drug monitoring (TDM). Among the four patients with IFD in the posaconazole group, three had plasma levels < 700 ng/mL, which could be associated with an increased risk of breakthrough fungal infections (Jang et al. 2010). One patient in this group died due to IFD. No cases of IFDs were observed in the micafungin group.

**Fig. 1:**
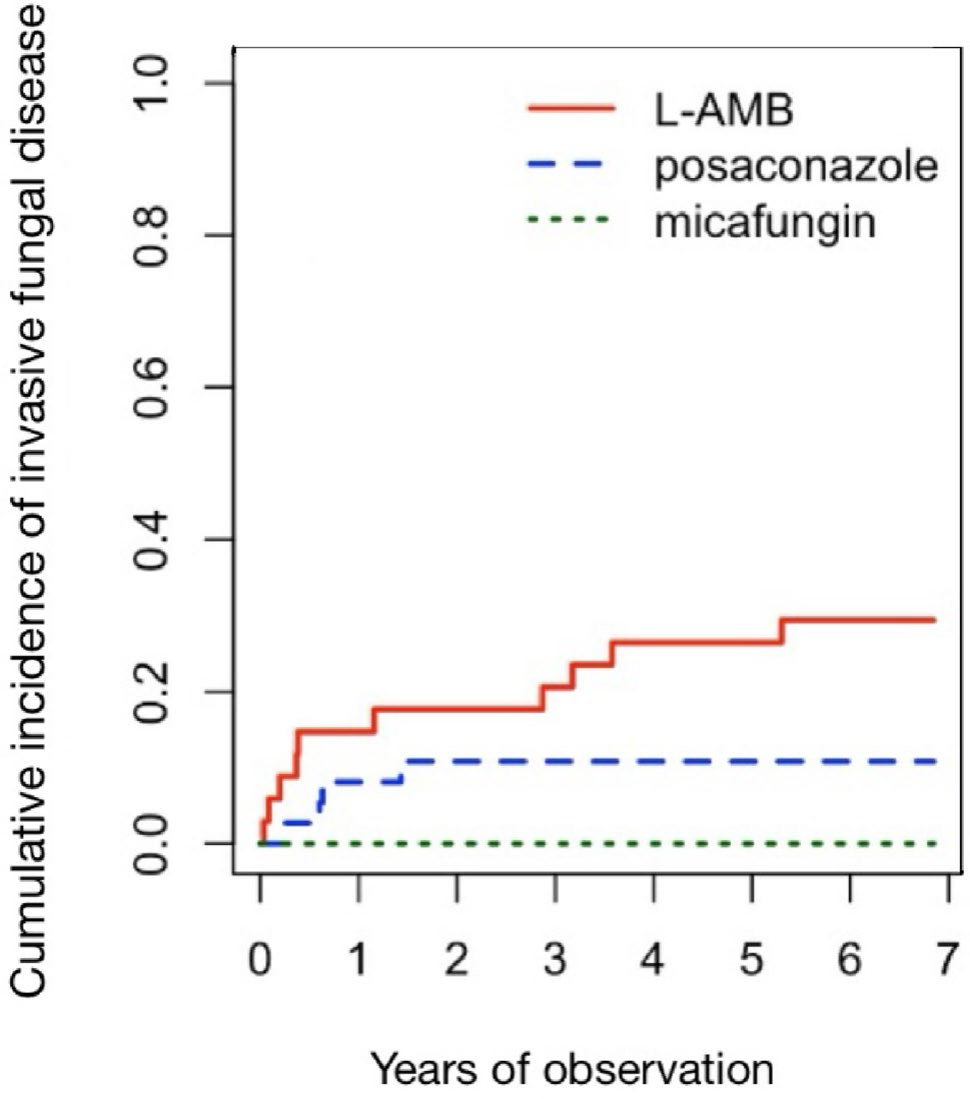
Cumulative incidence of invasive fungal disease in all patients, (*p* = 0.006)

Figure 1 shows the cumulative incidence of IFD across all three groups (L-AMB, posaconazole, micafungin), which differed significantly (Gray’s test, *p* = 0.006). Cumulative incidence of IFD was compared between the groups using the Gray test, competing events were not taken into account.

**Fig. 2:**
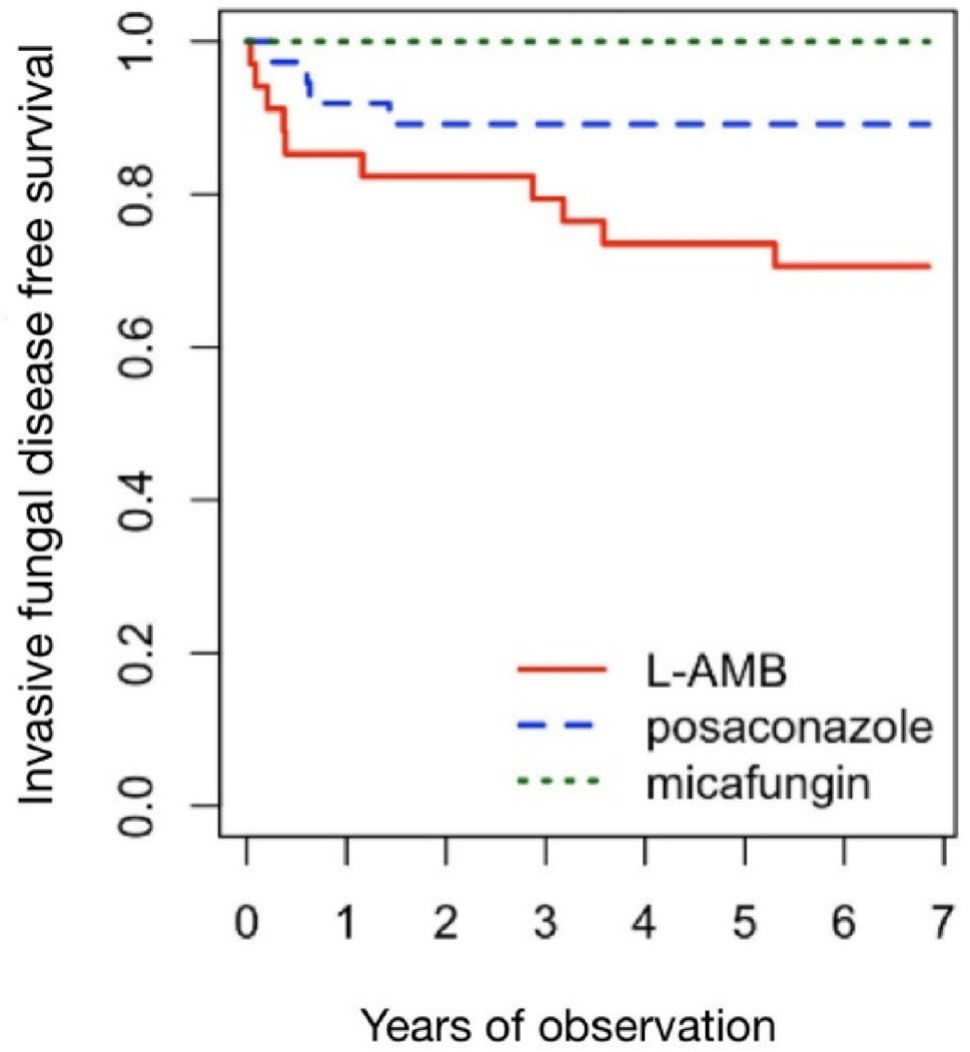
Invasive fungal disease-free survival of all patients (*p* = 0.005)

Figure 2 demonstrates the Kaplan–Meier curve estimate of IFD-free survival. IFD-free survival was 70.6% in the L-AMB group, 89.2% in the posaconazole group, and 100% in the micafungin group (*p* = 0.005).

In univariate Cox regression analysis (Table 4), antifungal prophylaxis with posaconazole or micafungin (compared to L-AMB) was significantly associated with a reduced risk of IFD (HR = 0.213; 95% CI 0.067–0.680; *p* = 0.009). In addition, age at diagnosis ≥ 10 years was also significantly associated with a higher risk of IFD (HR = 0.349; 95% CI 1.061–8.846; *p* = 0.039). A trend toward higher IFD risk was observed in patients with relapse (HR = 2.461; 95% CI 0.071–1.704; *p* = 0.193), although this did not reach statistical significance. Due to its potential clinical relevance and proximity to significance, this variable was considered for inclusion in the multivariate model. Other factors such as diagnosis (AML vs. ALL), HR-ALL vs. LR/IR-ALL, HSCT, and gender were not significantly associated with IFD-free survival.

**Table 4:** Univariate analysis of clinical risk factors associated with invasive fungal disease free survival

Variable	HR	95% CI	*p* value
Prophylaxis L-AMB vs. Posaconazole/Micafungin	0.213	0.067–0.680	**0.009**
Diagnosis (AML vs. ALL)	0.996	0.308–3.216	0.995
HR-ALL vs. LR/IR-ALL	0.990	0.250–3.910	0.988
Relapse	0.349	0.071–1.704	0.193
HSCT	0.721	0.229–2.273	0.577
Age at diagnosis below or above 10 years	3.064	1.061–8.846	**0.039**
Gender	0.679	0.225–2.051	0.492

Hazard Ratio, 95% confidence intervals and corresponding *p*-values are reported for each variable

*p*-values of less than 0.05 indicated statistical significance (in bold)

*ALL* acute lymphoblastic leukemia*, AML* acute myeloid leukemia*, CI* confidence interval*, HR* Hazard ratio*, HR-ALL* high risk ALL, *HSCT* hematopoietic stem cell transplantation*, LR/IR-ALL* low risk/intermediate risk ALL*, IFD* invasive fungal disease*, L-AMB* liposomal amphotericin B

### Multivariate analysis

In the multivariate Cox regression model for IFD-free survival, antifungal prophylaxis with posaconazole or micafungin (compared to L-AMB) remained independently associated with a significantly lower risk of invasive fungal disease (HR 0.248; 95% CI 0.0764–0.828; *p* = 0.023). In contrast, the occurrence of relapse (HR 0.523; 95% CI 0.140–1.956; *p* = 0.336) and age at diagnosis ≥ 10 years (HR 1.824; 95% CI 0.588–5.661; *p* = 0.298) were not statistically significant predictors in the multivariate model (Table 5).

**Table 5:** Multivariate analysis

Variable	IFD-free	
	HR (95% CI)	*P*
Prophylaxis L-AMB vs. Posaconazole/Micafungin	0.248 (0.764–0.828)	**0.023**
Relapse	0.523 (0.140–1.956)	0.336
Age at diagnosis below or above 10 years	1.824 (0.588–5.661)	0.298

Invasive fungal disease free survival and overall survival. Hazard ratios with 95% confidence intervals and corresponding *p*-values are reported for each variable

*p*-values of less than 0.05 indicated statistical significance (in bold)

CI confidence interval, *HR* Hazard ratio, *HSCT* hematopoietic stem cell transplantation, *IFD* invasive fungal disease, *L-AMB* liposomal amphotericin B

## Discussion

In this retrospective study, three different prophylactic antifungal regimens were compared in 95 pediatric patients diagnosed with ALL or AML undergoing chemotherapy at the Department of Pediatrics, Jena University Hospital, Jena, Germany, between January 2008 and December 2023. To our knowledge, this is the first study directly comparing the three antifungal agents: L-AMB, posaconazole, and micafungin for PAP in this specific patient population. A total of 14 cases of IFDs were identified, including four proven, six probable, and four possible cases. The incidence of breakthrough IFDs was highest in the L-AMB group (29.4%) followed by the posaconazole group (10.8%), and lowest in the micafungin group (0%). These findings suggest, that he choice of antifungal prophylaxis significantly impacts IFD-free survival and OS in pediatric patients with acute leukemia. Multivariate analysis confirmed that prophylaxis with either micafungin or posaconazole independently reduced the risk of IFD (HR = 0.248, *p* = 0.023) compared to L-AMB. In contrast, relapse and age at diagnosis ≥ 10 years were not significantly associated with IFD-free survival in the multivariate analysis. Notably, in the univariate analysis, patients with relapsed leukemia showed a trend toward increased IFD, although this did not reach statistical significance (*p* = 0.193). This trend may reflect a true difference that was underpowered in our cohort and warrants further investigation. These findings emphasize the importance of antifungal agent selection in pediatric leukemia patients and suggest that posaconazole and micafungin may offer superior protection against IFD compared to L-AMB, regardless of patient age, relapse status, or risk stratification.

L-AMB is an established antifungal agent approved for children aged 1 month to 18 years and shows potent activity against *Candida* and *Aspergillus* species. However, it is no longer recommended as the first-line prophylaxis due to its relatively high rates of breakthrough IFDs and the availability of more effective agents (Groll et al. 2021). Furthermore, there is poor evidence to recommend intravenous L-AMB prophylaxis specifically in patients with ALL (evidence level CI) (Stemler et al. 2023). Compared to conventional amphotericin B, L-AMB has significantly reduced nephrotoxicity and fewer infusion-related side effects (Stone et al. 2016). Despite its favorable safety profile, higher doses (above 5 mg/kg/day) have been associated with an increased occurrence of hypokalemia and infusion-related vomiting (Maertens et al. 2022). Interestingly, Cornely et al. (2017) found no significant difference in IFD incidence when comparing L-AMB at 5 mg/kg per week with placebo recipients. In line with our findings, this data suggest limited efficacy of L-AMB in certain patient populations, further supporting its role as a second-line agent when other prophylactic drugs are contraindicated.

Posaconazole is an FDA-approved antifungal agent for use in children aged ≥ 2 years, with broad-spectrum activity against a wide range of medically relevant yeasts and molds including many rare fungal pathogens (Bury et al. 2021). Available formulations include oral suspension, delayed-release tablets and intravenous (IV) solutions (Groll et al. 2020). Its extensive spectrum of activity makes it a first-line option for antifungal prophylaxis in many centers (Groll et al. 2024). Posaconazole is generally well tolerated, with gastrointestinal disturbances, headaches, and elevated liver function tests being the most commonly reported adverse effects (Groll et al. 2024). However, it is a potent inhibitor of the cytochrome P450 enzyme CYP3A4, which can result in significant drug-drug interactions (DDIs). In particular, coadministration with vinca alkaloids, such as vincristine, has been associated with elevated plasma concentrations of the chemotherapeutic agent, increasing the risk of severe neurotoxicity (Moriyama et al. 2012). Consequently, the concurrent use of posaconazole and vinca alkaloids is generally contraindicated. For patients with neutropenia, posaconazole is strongly recommended as an antifungal prophylactic agent (AI recommendation), with evidence from a large randomized controlled trial that utilized the oral suspension formulation (Mellinghoff et al. 2018). In our study, posaconazole was associated with a relatively low rate of breakthrough IFDs (10.8%), encompassing proven, probable, and possible infections. Similarly, a recent South Korean study reported an incidence of 6.8% IFDs, comprising 2.5% proven or probable cases and 4.3% possible cases (Yang et al. 2021). TDM plays a critical role in optimizing the efficacy of posaconazole, as inadequate plasma levels are linked to higher rates of breakthrough infections. Future studies should explore the potential of TDM to enhance clinical outcomes in this patient population (Liszka et al. 2025).

Our results suggest that micafungin may be more effective in preventing IFDs in patients with acute leukemia. Our observation is supported by a recent study conducted by Bury et al. (2024), which reported a significant reduction in the incidence of proven and probable *Aspergillus* infections in patients with ALL treated with micafungin, particularly during the induction phase. Similarly, Venton et al. (2016) demonstrated an incidence of 0.0% IFDs in patients diagnosed with AML and micafungin as prophylaxis. In contrast, Lopez-Sanchez et al. (2020) reported a 4.4% incidence of proven or probable IFDs in patients undergoing HSCT who received micafungin prophylaxis in a retrospective multicenter observational study. Despite these promising results, data on micafungin as a prophylactic agent in pediatric leukemia remain limited. Additional studies with larger sample sizes are needed to validate its efficacy. The superior outcomes observed with micafungin and posaconazole may be explained by their broad antifungal spectrum, more favorable toxicity profiles, and better tolerability in pediatric patients undergoing intensive chemotherapy.

Several limitations should be acknowledged. Firstly, fungal susceptibility testing was not routinely performed in this cohort, which limits our ability to assess potential antifungal resistance. Additionally, although antifungal pharmacokinetics and immune reconstitution may contribute to higher IFD risk in patients aged ≥ 10 years, it is also possible that this association reflects a higher rate of relapse within this age group. However, in our study, treatment intensity was protocol-driven and not directly based on age. Although differences in IFD risk across distinct chemotherapy phases, particularly induction and reinduction, are clinically relevant, subgroup analyses comparing the efficacy of the three prophylactic regimens during specific treatment phases were not feasible due to limited case numbers per group. Moreover, the retrospective design and long study period contributed to heterogeneity in treatment protocols, diagnostic modalities, and risk stratification criteria. We acknowledge that the underlying leukemia risk group could impact the risk of IFD. Future prospective studies should aim to systematically evaluate this factor.

Furthermore, we recognize that the micafungin group included a higher proportion of patients with lower expected IFD risk, such as those without HSCT or with lower-risk leukemia subtypes. This may have influenced the favorable outcomes observed in this group. Although we attempted to address this through multivariate modeling, residual confounding cannot be fully excluded and represents a limitation of our study.

Our findings highlight the complexity of risk assessment and emphasize the need for individualized prophylaxis strategies individualized to patient- and disease-specific factors. Detailed subgroup analyses, such as those proposed by Lernbecher et al. (2019), may provide further insights into patient-specific risks of individualized risk assessment and prophylaxis selection in patients with acute leukemia. Our data suggest that not only disease characteristics and patient age but also the choice of antifungal agent has a substantial impact on patient outcomes.

Despite these limitations, our findings provide real-world data on the effectiveness of three antifungal prophylaxis strategies in pediatric patients with acute leukemia. In conclusion, we observed a significant difference between the three antifungal regimens L-AMB, posaconazole and micafungin in their efficacy. Micafungin had the lowest IFD breakthrough rate. To confirm the results and to refined guideline recommendations, multicenter clinical studies including more patients would be needed in the future.

## Data Availability

No datasets were generated or analysed during the current study.
